# Intranasal insulin administration reduces oxidative stress‐induced damage and improves mitochondrial bioenergetics in Alzheimer disease

**DOI:** 10.1002/alz.087320

**Published:** 2025-01-03

**Authors:** Antonella Tramutola, Sara Pagnotta, Fabio Di Domenico, Lucrezia Romana Rolfi, Marzia Perluigi, Eugenio Barone

**Affiliations:** ^1^ Sapienza University of Rome, Rome, Rome Italy; ^2^ Sapienza University of Rome, Rome Italy; ^3^ Department of Biochemical Science, Sapienza University of Rome, Rome Italy

## Abstract

**Background:**

Brain insulin resistance (bIR) heavily impacts on the core pathological processes of aging and Alzheimer disease (AD) since insulin regulates brain metabolism and cognitive functions. A close link among bIR, oxidative stress (OS) and mitochondrial defects exists, that contributes to brain dysfunctions observed in AD. Intriguingly, several studies suggest that intranasal insulin treatment (INI) enhances cognitive performance and reduced AD neuropathology both in humans and murine models of AD. We focused on the interplay between OS and bIR, by testing the hypothesis that rescuing brain insulin signaling activation by INI results in improved mitochondrial functions and reduced OS‐induced damage to proteins in a mouse model of AD (3xTg‐AD).

**Method:**

12‐month‐old 3 × Tg‐AD and wild‐type (non‐Tg) mice were treated with INI (2 UI) or vehicle (saline) every other day for 2 months. Insulin signaling pathway and OS marker levels, i.e., PC, 4‐HNE and 3‐NT were evaluated in the frontal cortex. Then, due to the link between bIR and nitrosative stress, a redox proteomics approach was used to identify specific protein targets of 3‐NT modifications. Mitochondrial functions were evaluated by measuring mitochondrial complexes (OXPHOS) and activities in all experimental groups.

**Result:**

INI administration improved insulin signaling and reduced OS levels in 3xTg‐AD mice. In particular, a consistent reduction of 3‐NT levels was observed. Redox proteomics allowed to identify several proteins with reduced 3‐NT modifications, that belong to key pathways, such as protein degradation and energy metabolism, known to be involved in the progression of AD. Remarkably, reduced 3‐NT levels on mitochondrial proteins were responsible for an improvement of mitochondrial activity and brain energy metabolism.

**Conclusion:**

We propose that INI represents a promising approach to reduce OS‐induced damage to proteins and restore mitochondrial bioenergetics in AD brain.

